# Fatal co-infection by multiple pathogens in an indigenous woman with autoimmune hemolytic anemia and tuberculosis: a case report

**DOI:** 10.1186/s12879-024-09557-w

**Published:** 2024-06-27

**Authors:** Bryan Tabares, Alisson Dayana Sarmiento-Suárez, Óscar Gil, Juan Camilo Hernández-Pabón, Carolina Firacative

**Affiliations:** 1 Unidad de Extensión Hospitalaria, Hospital Universitario Mayor Méderi, Bogota, Colombia; 2 Unidad Hemato-Oncología, Hospital Universitario Mayor Méderi, Bogota, Colombia; 3 Group MICROS Research Incubator, School of Medicine and Health Sciences, Universidad del Rosario, Bogota, Colombia; 4 Studies in Translational Microbiology and Emerging Diseases (MICROS) Research Group, School of Medicine and Health Sciences, Universidad del Rosario, Bogota, Colombia

**Keywords:** Autoimmune hemolytic anemia, Bacterial infections, Invasive fungal infections, Drug-resistance, Indigenous, Tuberculosis

## Abstract

**Background:**

Tuberculosis (TB), one of the leading causes of death worldwide, has a higher incidence among indigenous people. Albeit uncommon, autoimmune hemolytic anemia (AIHA) has been deemed a risk condition to develop mycobacterial infection, as a result of the immunosuppressive treatments. TB, in turn, can be a predisposing factor for secondary infections.

**Case presentation:**

Here we present a case of a 28-year-old indigenous woman from Colombia, previously diagnosed with AIHA and pulmonary TB. Despite various treatments, therapies and medical interventions, the patient died after severe medullary aplasia of multiple causes, including secondary myelotoxicity by immunosuppressive therapy and secondary disseminated infections, underlining infection by *Staphylococcus aureus, Klebsiella pneumoniae* and *Candida glabrata*, which were identified as drug-resistant microorganisms. Together, this led to significant clinical complications. Invasive aspergillosis was diagnosed at autopsy.

**Conclusions:**

This report presents a rarely finding of AIHA followed by TB, and highlights the great challenges of dealing with co-infections, particularly by drug resistant pathogens. It also aims to spur governments and public health authorities to focus attention in the prevention, screening and management of TB, especially among vulnerable communities, such as indigenous people.

## Background

Tuberculosis (TB), caused by the bacillus *Mycobacterium tuberculosis*, is one of the leading causes of death worldwide. In 2021, TB accounted for an estimated 1.6 million deaths out of the 10.6 million people that fell ill with this infection [1]. Among indigenous people, particularly, the incidence and mortality of TB are generally higher, even though the burden of the disease in these communities is not always known [1, 2]. Chronic illnesses, malnutrition, overcrowding, inequity and poor healthcare access of indigenous populations, increase the risk of TB transmission and progression to active disease, which is in turn associated with delayed diagnosis and treatment [3]. Uncommon disorders, such as autoimmune hemolytic anemia (AIHA), characterized by the production of autoantibodies directed against red blood cells, can also increase the risk of TB reactivation, mainly because of the use of high or prolonged doses of steroids, which impair the immune response [4–6]. Another determinant of the severity of the disease and the outcome of patients with TB are secondary infections, not only because additional diagnostic tests and treatments are required, but also because, currently, various species of bacteria and fungi are characterized by intrinsic or acquired resistance to one or more antimicrobials, which hinders treatment and leads to important clinical complications that further aggravates the patient condition [7, 8]. Herein, we describe a fatal case of an indigenous woman previously diagnosed with AIHA and pulmonary TB who acquired viral, bacterial and fungal secondary infections following hospitalization. Notoriously, some bacterial and fungal species concurring with TB were identified as drug-resistant.

## Case presentation

An indigenous 28-year-old woman from Guaviare, a southern-central region state of Colombia originally inhabited by the indigenous Nukak people, presented to a local hospital with a 15-day history of fatigue, shortness of breath and tensional headache. She had a 2-year medical history of AIHA, due to mixed autoantibodies of unknown etiology, with a daily oral treatment of azathioprine (50 mg) and prednisolone (25 mg) since diagnosis. In addition, an extra-institutional report indicated that 10 months ago the patient was diagnosed with pulmonary TB following positive serial bacilloscopy and the isolation of *M. tuberculosis*, resistant to isoniazid, from sputum culture. During this episode of TB, the patient did not receive any treatment owing to limited access to antimycobacterial agents. It was also unknown if screening for latent TB was done before the diagnosis of AIHA. She did not report fever, weight loss, cough, or any other symptoms. Because of her medical history, a complete blood count and a hemolytic anemia workup were performed, finding a hemolytic crisis (hemoglobin 4.9 g/dL, lactate dehydrogenase (LDH) > 1000 U/L and indirect hyperbilirubinemia) but normal leukocytes and platelets counts. Due to her condition, she received a blood transfusion and high-dose intravenous methylprednisolone without any response. Therefore, she was referred to a fourth level hospital for integrated clinical management.

At admission to our hospital, cyanocobalamin and folic acid deficiency, HIV infection, hepatitis B and C, syphilis, and hemolytic anemia secondary to autoimmune diseases such as systemic lupus erythematosus, were ruled out. A chest radiography did not show any apparent lung abnormalities. At day 5, the patient presented hemodynamic instability and was admitted to the intensive care unit (ICU). Considering the null response to methylprednisolone, the worsening of anemia and the persistence of an active hemolytic profile, another blood transfusion and management with four doses of anti-CD20 (rituximab), were indicated. Unfortunately, refractoriness to this therapy was reported, therefore management with a four-dose cyclophosphamide (CYC) scheme was added. Due to hematological toxicity manifested as pancytopenia, the last CYC dose was not administered.

During her ICU stay, the patient developed several secondary infectious complications, starting with oral and vulvovaginal mucositis and multiple-organism bacteremia (MOB) with isolation, from blood, of *Escherichia coli*, methicillin resistant *Staphylococcus aureus* (MRSA) and *Enterococcus faecium* vancomycin susceptible. Consequently, she was indicated with multiple antibiotic schemes including ceftaroline fosamil and ceftazidime-avibactam plus aztreonam combination.

Despite multiple antibacterial treatments and even though her LDH and total bilirubin levels normalized, the patient persisted with febrile neutropenia and deeper cytopenia. Considering that the patient was not receiving any myelosuppression drugs, there was suspicion of bone marrow infection by atypical microorganisms. Therefore, bone marrow biopsy and myelocultures were ordered to determine the presence of pathogens in this tissue or a hidden neoplasia. Pathological investigations reported a hypocellular (10%), subcortical and fragmented bone marrow without infiltration. Iron, Grocott methenamine-silver and periodic acid-Schiff stains, as well as Rose Bengal test and myelocultures were negative. Nevertheless, given the persistence of fever and bone marrow aplasia, antimicrobial coverage against intracellular pathogens that could compromise bone marrow function, was indicated. This combined therapy included clindamycin, doxycycline, ivermectin and azithromycin. Specific tests for parvovirus B19 and malaria were found negative. PCR for TB of a bone marrow aspirate was negative too. Even though herpes simplex virus 2 IgG and cytomegalovirus viral loads were detected in blood, neither clinical nor symptomatic improvement of the patient was observed after treatment with acyclovir and ganciclovir.

While conducting additional studies, the patient presented gross hematuria. A urinalysis reported proteinuria, hematuria, glycosuria, and active sediment. A computerized tomography (CT) scan of the urinary tract revealed bilateral nephromegaly. Additionally, nephrotic-range proteinuria was documented, with indication for renal biopsy, which was deferred due to the severe aplasia. Considering these findings, the patient’s origin, and the medical history, there was a high suspicion of genitourinary TB. However, a PCR for TB in urine gave a negative result.

**Fig. 1:**
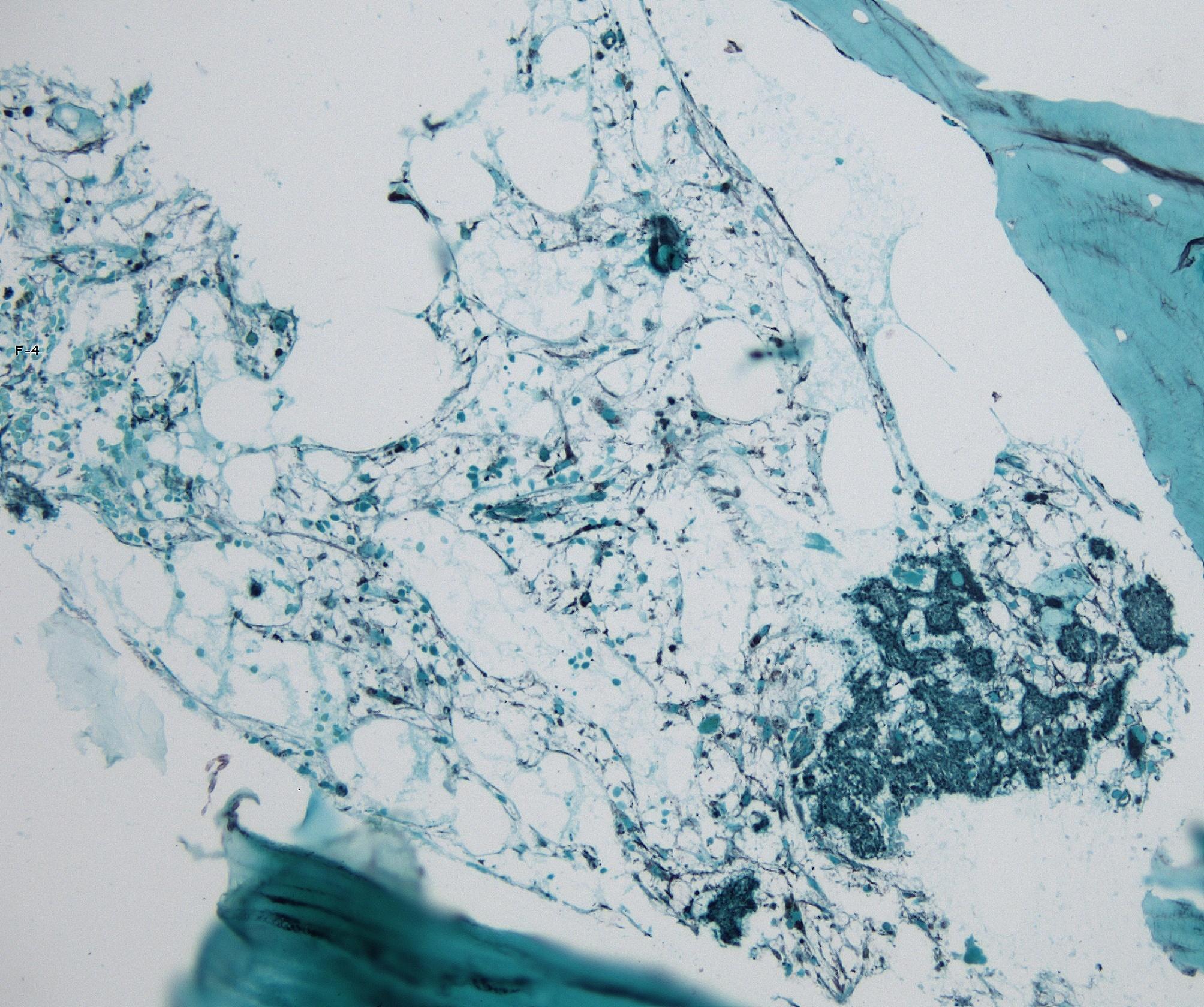
Microphotograph of the bone marrow biopsy showing low cellularity and scarce structures suggestive of blastoconidia. Grocott-Gomori methenamine silver staining. Original magnification 20X

As the patient persisted with severe bone marrow aplasia associated with complications by MOB, a second bone marrow biopsy was performed as well as myelocultures and hemocultures, to ruled out deep mycoses. At first, bone marrow biopsy pathology revealed suggestive blastoconidia (Fig. 1), therefore, liposomal amphotericin B was indicated with poor response. Hematolymphoid neoplasms was not observed. Afterwards, both from bone marrow and blood, *Candida glabrata* was isolated, switching the antifungal treatment to caspofungin. *Klebsiella pneumoniae* carbapenemase (KPC)-producing was also recovered from bone marrow and blood samples. A PCR and Ziehl-Neelsen stain for *M. tuberculosis* in bone marrow were reported negative.

**Fig. 2:**
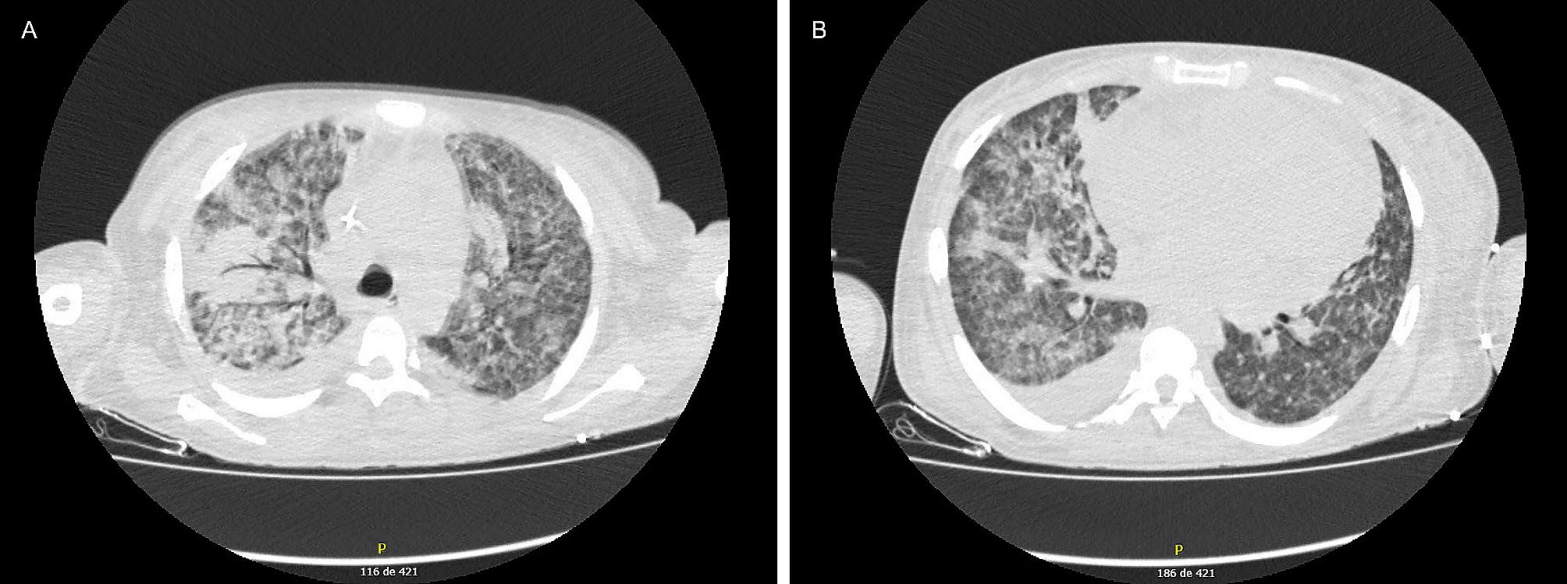
Computer tomography scan of the chest of a 28-year-old indigenous woman with tuberculosis, autoimmune hemolytic anemia and multiple pathogen co-infections. (**A**) Multifocal alveolar opacities. (**B**) Pleural effusion, more extensive on the right side, and cardiomegaly

A CT scan of the lungs revealed alveolar opacities, pleural effusion and cardiomegaly (Fig. 2). Considering the medical history, TB tetraconjugated treatment plus moxifloxacin was indicated. Given the high complexity of this case, a medical board, including critical care, infectious diseases, hematology and pneumology specialists, was held. It concluded that the medullary aplasia has multiple causes, being secondary to myelotoxicity by immunosuppressive therapy, due to AHIA, and infectious diseases including TB, with pulmonary involvement, as well as MOB by antibiotic-resistant bacteria, and bone marrow mycosis and candidemia by *C. glabrata*, which was resistant to caspofungin, as established later (MIC of 64 µg/ml).

Despite polymicrobial treatment and multiple red blood cell and platelet transfusions, due to anemia and thrombocytopenia, the patient was in a poor general condition, tachycardic, polypneic and required continuous positive airway pressure (CPAP) therapy. In addition, pancytopenia and neutropenia persisted, in spite of the administration of filgrastim. Given that the severe aplasia could not be resolved, the patient presented hemodynamic and respiratory failure, with indication for invasive mechanical ventilation. However, she refused invasive procedures including cardiopulmonary resuscitation or intubation. At day 114, the patient died because of acute respiratory failure. Autopsy revealed the presence of 45-degree angled, branching hyphae, suggestive of *Aspergillus*, in lungs and kidneys. After her death, a relative of the patient informed that she was a close contact of two people with TB, from whom one died of this mycobacterial infection.

## Discussion and conclusion

There are great challenges when dealing with co-infections by multiple pathogens, since they are more complex to diagnose and treat, and have a worse outcome than either infection on its own. Moreover, some complications are not always borne in mind and these remain undiagnosed until autopsy. While tuberculosis and bacterial or fungal co-infections have been more commonly described in HIV/AIDS patients, they can also occur in patients with preserved immunity [9, 10]. Particularly in indigenous people, albeit HIV-seronegative but with induced immunosuppression, the occurrence of TB together with other multiple infectious diseases must be always considered, including invasive aspergillosis for which TB is a common underlying condition [11, 12]. In Colombia, not only the incidence of TB is 2.5 times higher among indigenous communities compared with the general population, but in these people the risk to develop TB-associated opportunistic infections is also higher, considering the context of poverty, malnutrition, underlying medical conditions, difficulties of geographic access, delay in seeking for medical care and the preexisting lung disease per se [2, 13, 14].

Added to the difficulties in diagnosing and treating co-infections among TB patients, are the diverse effects on the hematopoietic system during the course of mycobacterial infection. Not only common hematologic disorders seen in TB patients such as anemia, thrombocytosis and white blood cell disorders can be associated with increased mortality and poor response to antituberculous treatment, but also atypical underlying conditions such as AIHA [15, 16]. In patients with this decompensated acquired hemolysis, the high-dose and prolonged treatment with steroids is associated with infectious complications, such as TB and other opportunistic infections [5, 6, 10, 17].

Importantly, screening and preventive treatment for TB are recommended in people with high-risk of infection, such as indigenous populations, or in patients undergoing immunosuppressive therapy, such as those with AIHA [3, 5, 18]. However, in our country, particularly in remote areas, there are clear barriers regarding the access, use and implementation of methods targeting latent TB infection, which impedes performing TB risk assessment, early diagnosis and correct management of patients, therefore preventing a substantial reduction in TB incidence and mortality [13, 19].

The threats posed by antimicrobial resistance further increase the difficulties of managing multiple pathogen infections in TB patients. In the case reported herein, not only resistant Gram-positive and Gram-negative bacteria were found concomitantly with an untreated isoniazid resistant *M. tuberculosis*, but also with a caspofungin resistant *C. glabrata*. Treatment failure amplifies the severity of the disease and deteriorate the patient’s condition, leading to a poor prognosis and death [7, 8, 18].

Considering that indigenous people often refer to hospital when they are already in a very bad condition, or when they have used traditional medicine and self-medications, prompt diagnoses and accurate treatments are essential. Clinicians, in addition, should have a low threshold for suspecting hematological disorders as risk factors in these patients, as well as co-infections by resistant pathogens, which warrant special attention. Lastly, governments and public health authorities must focus research in prevention, screening and management of TB among indigenous communities, working towards reducing the ongoing transmission in these populations by lessening health inequalities and other mitigating factors that support and perpetuate poor health.

To our best knowledge, this is the first report about an indigenous woman, HIV-seronegative, who presented TB, which was very likely attributed to AIHA and inherent factors of indigenous populations. Unfortunately, the patient died by multiple complications including disseminated co-infections caused by various pathogens, some of them drug resistant such as MRSA, KPC-producing *K. pneumoniae* and *C. glabrata* caspofungin-resistant.

## Data Availability

All data generated or analyzed during this study are included in this published article.

## Abbreviations

AIDS: Acquired immune deficiency syndrome
AIHA: Autoimmune hemolytic anemia
CPAP: continuous positive airway pressure
CT: Computerized tomography
CYC: cyclophosphamide
HIV: Human immunodeficiency virus
ICU: Intensive care unit
KPC: Klebsiella pneumoniae carbapenemase
LDH: Lactate dehydrogenase
MIC: Minimal inhibitory concentration
MOB: Multiple-organism bacteremia
MRSA: Methicillin resistant Staphylococcus aureus
PCR: Polymerase chain reaction
TB: Tuberculosis
